# Appetitive Aggression and Adverse Childhood Experiences Shape Violent Behavior in Females Formerly Associated with Combat

**DOI:** 10.3389/fpsyg.2015.01756

**Published:** 2015-11-17

**Authors:** Mareike Augsburger, Danie Meyer-Parlapanis, Manassé Bambonye, Thomas Elbert, Anselm Crombach

**Affiliations:** ^1^Department of Psychology, University of KonstanzKonstanz, Germany; ^2^Department of Psychology, University LumièreBujumbura, Burundi

**Keywords:** posttraumatic stress disorder (PTSD), trauma, childhood maltreatment, violence, aggression, female combatant, Burundi, post-conflict country

## Abstract

This study investigated the impact of violent experiences during childhood, posttraumatic stress disorder (PTSD) and appetitive aggression on everyday violent behavior in Burundian females with varying participation in war. Moreover, group differences in trauma-related and aggression variables were expected. Appetitive aggression describes the perception of violence perpetration as fascinating and appealing and is a common phenomenon in former combatants. Semi-structured interviews were conducted with 158 females, either former combatants, supporters of armed forces or civilians during the civil war in Burundi. The PTSD Symptom Scale Interview was used to assess PTSD symptom severity, the Appetitive Aggression Scale to measure appetitive aggression and the Domestic and Community Violence Checklist to assess both childhood maltreatment and recent aggressive behavior. Former combatants had experienced more traumatic events, perpetrated more violence and reported higher levels of appetitive aggression than supporters and civilians. They also suffered more severely from PTSD symptoms than civilians but not than supporters. The groups did not differ regarding childhood maltreatment. Both appetitive aggression and childhood violence predicted ongoing aggressive behavior, whereas the latter outperformed PTSD symptom severity. These findings support current research showing that adverse childhood experiences and a positive attitude toward aggression serve as the basis for aggressive behavior and promote an ongoing cycle of violence in post-conflict regions. Female members of armed groups are in need of demobilization procedures including trauma-related care and interventions addressing appetitive aggression.

## Introduction

Even several years after the establishment of peace, the inhabitants in post-conflict regions still struggle with the aftermath of war. Poverty, poor health, lack of security, and high rates of violence pose major challenges to both the individual and the overarching society (Saile et al., 2014). War-affected individuals often contend with significant mental health complications. In line with the *building block effect*, i.e., mental ill-health due to cumulative exposure to traumatic stressors (Neuner et al., 2004), prevalence rates of posttraumatic stress disorder (PTSD) in war-affected populations are severely elevated (e.g., Steel et al., 2002; Karunakara et al., 2004; Nandi et al., 2015). In addition, a growing body of research demonstrates a link between traumatization and enhanced aggressive behavior in military personnel that continues after deployment (Byrne and Riggs, 1996; Morland et al., 2012; MacManus et al., 2013). For some individuals returning from combat, PTSD symptoms, such as hyperarousal and angry outbursts, are exhibited in violent behavior (Galovski and Lyons, 2004; Orth and Wieland, 2006).

Though high rates of PTSD are present among former combatants, hyperarousal cannot fully account for the high prevalence of violence observed in post-war societies. In fact, Elbert et al. (2010) postulated that active combatants and child soldiers might perceive the perpetration of brutal aggressive acts as appealing and intrinsically rewarding. This perception of violence as exciting, i.e., appetitive aggression, has been reported by a variety of combatants in different post-conflict regions. Several studies suggest that appetitive aggression helps combatants maintain functionality and cope with trauma-related mental health symptoms in life threatening and violent environments (e.g., Hecker et al., 2012; Weierstall et al., 2013a,b). While developing appetitive aggression in such adverse environments seems to be beneficial in order to regain feelings of power and control, it most likely also enhances the likelihood of violent behavior (Crombach and Elbert, 2015). Moreover, high levels of appetitive aggression impede the successful reintegration of former combatants (Maedl et al., 2010).

Beyond the impact of PTSD and appetitive aggression in explaining ongoing violence in post-conflict regions, research has also focused on the effect of childhood maltreatment. According to the *cycle of violence* hypothesis (Widom, 1989), adverse childhood experiences culminate in aggressive behavior toward one’s own children – thus producing an ongoing climate of violence in families. This trans-generational effect of childhood maltreatment was also observed in Sub-Saharan African post-conflict regions. Rwandan parents with histories of childhood abuse had an elevated risk of perpetrating violence against their own children (Rieder and Elbert, 2013; Roth et al., 2014). However, the impact of childhood maltreatment and trauma-related disorders on abusive child-rearing practices is not yet fully understood (Pears and Capaldi, 2001; Catani, 2010).

The majority of studies focusing on the relationship between combat exposure and ongoing violence in post-conflict regions almost exclusively focused on male combatants or soldiers (McKay and Mazurana, 2004; Coulter et al., 2008; Herrmann and Palmieri, 2010). However, studies indicate that females in conflict regions are also active agents of warfare and make up a proportion to 30% of members of armed groups (Brett, 2002; Mazurana, 2004). Girl soldiers were part of fighting forces in 55 countries, 38 of these were involved in internal wars between 1990 and 2003 (McKay and Mazurana, 2004). Females cover a variety of tasks, ranging from supportive, caretaking roles (e.g., cooking or washing) to performing as armed combatants (Mazurana and Carlson, 2004; Coulter et al., 2008; Annan et al., 2009). Some also hold central commanding roles, having achieved high-ranking military positions and authority (Mazurana, 2004; Coulter et al., 2008). For many, membership in an armed group is accompanied by an expansion of traditional gender roles and thereby new possibilities (Coulter et al., 2008).

Though the number of quantitative studies about women or girls at war has been increasing, little is known about the challenges with which female, former members of armed groups contend post-war. Only a minority have been formally included in disarmament, demobilization, and reintegration processes (Schauer and Elbert, 2010). In a review of US soldiers, female active-duty service members were found to be at the same risk for developing PTSD as their male counterparts (Chaumba and Bride, 2010). In a survey about youth in Uganda, girls abducted by Lord’s Resistance Army (LRA) reported 20% higher rates of psychological distress compared to female non-abductees, even years after their return home (Annan et al., 2011).

In probably all cultures, females are assumed to be less aggressive than males (Richardson, 2005; Stockley and Campbell, 2013). However, sex differences vary depending on the social context and different forms of aggression (Archer, 2004; Campbell, 2006). From an evolutionary point of view, it has been argued that males have been more involved in competition regarding social status, wealth, and sexual partners, and that for them aggressive behavior might be a promising strategy (Wilson et al., 2002). Campbell (2013) argues, due to their evolutionary higher involvement in raising children females prefer strategies with lower risk to get injured such as indirect forms of aggression in order to not jeopardize reproductive success. In contrast, Richardson and Hammock (2007) emphasize the impact of gender role expectations, as the traditional role model of femininity is inconsistent with aggressive acts. Concerning appetitive aggression in females, studies are limited and inconsistent. In a sample of Rwandan genocide perpetrators, females reported lower levels than males (Weierstall et al., 2011). In contrast, no gender effect in the prediction of appetitive aggression was revealed in a sample of Columbian ex-combatants (Weierstall et al., 2013a). Meyer-Parlapanis et al. (submitted) found that females when having experienced similar combat-related events can develop levels comparable to males. Aiming to further strengthen the knowledge about the challenges female ex-combatants face post-war, particularly in Sub-Saharan African post-conflict regions, the present study was conducted.

Burundi was selected for data collection owing to its continued struggle in the aftermath of a long-lasting civil war. It is a small but densely populated state in the African Great Lakes Region that has suffered a long history of ethnic violent conflicts. In 1993 the conflict escalated into a civil war between the Tutsi-dominated army and armed Hutu rebel groups (United States Institute of Peace, 2002). Throughout this conflict over 300,000 people, mostly civilians, were killed. The war ended in 2006 (Uvin, 2009), and the last demobilizations of rebel members officially took place in 2009 (World Bank, 2009). Today, the country continues to grapple with high levels of violence. The recent violent outbursts in response to political elections exemplify the sustained fragility of peace in Burundi (United Nations, 2015).

With the present study it was aimed to assess how females in settings like Burundi cope with adverse experiences made throughout their lives. Female, former members of armed groups were compared to civilians who had never been active agents in the civil war. Former rebels were further allocated to two groups: combatants having participated in active fighting or supporters having been only involved in supportive, non-military tasks.

We investigated differences in exposure to childhood violence and traumatic events, as well as the perpetration of violent acts and their consequences for mental health in terms of PTSD and appetitive aggression. Moreover, we assessed predictors of low-threshold daily aggressive behavior to gain insight into the cycle of violence. The highest levels of exposure to both traumatic and perpetrated events were expected within former members of armed groups as well as high levels of appetitive aggression in former combatants due to their combat experience. A similar pattern was assumed to hold true for PTSD symptoms because of the building block effect. Finally, threshold changes in aggressive behavior were expected between the groups. It was hypothesized that high levels of appetitive aggression and PTSD contribute to perpetrating more recent aggressive acts. Furthermore, the impact of experienced childhood maltreatment on the assumed relationships between PTSD, appetitive aggression and violent behavior was of interest.

## Materials and Methods

### Participants

Semi-structured diagnostic interviews were conducted with 158 women in Burundi who had either been former combatants (*n* = 54), supporters of armed groups without involvement in fighting (*n* = 50), or civilians (serving as control group, *n* = 54). One former combatant was excluded prior to data analysis due to discrepancies in the information provided. Demographics of the three groups are shown in **Table 1**.

**Table 1:** Participant demographics and military involvement.

	Fighter (*n* = 53)	Supporter (*n* = 50)	Control (*n* = 54)
**Demographics**			
Age, years, *M* (*SD*) [*range*]	30.83 (7.18) [20–55]	32.94 (9.55) [18–58]	30.45 (7.76) [19–58]
Education, years, *M* (*SD*) [*range*]	6.62 (3.78) [0–13]	4.78 (3.58) [0–13]	5.35 (4.74) [0–16]
Children, No., *M* (*SD*) [*range*]	2.51 (2.03) [0–7]	3.02 (2.36) [0–10]	3.15 (2.2) [0–8]
**Variables regarding membership in armed groups**			
Child soldier, No., *(%)*	31 (59)	26 (52)	NA
Joined by force, No., *(%)*	18 (34)	16 (33)	NA
Duration, years, *M* (*SD*) [*range*]	4.77 (2.23) [2–11]	4.76 (3.00) [1–15]	NA

*M, mean; SD, standard deviation, No., number, NA, not applicable.*

Respective statistical tests indicated no significant differences between the groups in age, number of children, education, and working situation (all *p* ≥ 0.07). Former combatants and supporters did not differ regarding military variables (all *p* ≥ 0.37).

### Procedure

Data collection was carried out in fall 2014 in Bujumbura, Burundi. Former armed group members were invited to the study with the help of a local contact person from an official national veteran association. Female civilians inhabiting the same neighborhoods as the former members of armed groups were invited to participate as controls. A mixed team of experienced clinical psychologists from the University of Konstanz and trained local psychology students conducted the interviews. The latter either worked as translators (English/French – Kirundi) or performed interviews on their own following intensive training. They had gathered extensive experience in previous projects and were closely supervised to guarantee high interviewer reliability. Each interview lasted about 2–3 h and took place in either the rooms of the Centre for Mental Health (Centre Akabanga) or at a military training compound (Camp Muha) in Bujumbura, both provided by the Burundian army. Interviewers ensured that the interviews were conducted privately. Participants received 10,000 BIF (approximately 5€) for compensation and a refund of transport costs. In addition, respondents were offered to participate in another study (not reported here). The Ethical Review Boards of both the University of Konstanz and the University Lumière of Bujumbura approved the study. All participants provided informed consent.

### Measures

All instruments were translated and blindly back translated from a validated English or French version into Kirundi. Differences in meaning were discussed with both translators and a team of local psychologists until a consensus was reached.

#### Socio-demographics and Military Involvement

Participants were asked for age, level of education, working situation, and number of children. When applicable, details about participation in the rebel movement were asked (year of and age at entry, forced or voluntary joining, duration spent in the armed group).

#### Traumatic Event Types

A 20-item event list that has been applied in different contexts with populations affected by violent conflicts (Neuner et al., 2004; Nandi et al., 2015) was used to assess lifetime traumatic load. Events from the Posttraumatic Diagnostic Scale (Foa et al., 1997) were incorporated as well as different war-related witnessed and self-experienced events (e.g., being attacked). It was asked whether or not certain events had been experienced. Thus, items were coded dichotomously with 0 (no) or 1 (yes). Event types were summed up to measure the total traumatic load.

#### Perpetrated Event Types

To assess lifetime self-committed violence, 15 different types of perpetrated violence (e.g., committed assaults, mutilation) were assessed. The checklist has been applied in different combatant samples (Weierstall and Elbert, 2011). Coding of items was the same as for traumatic event types.

#### Childhood Violence

Exposure to violence during childhood was assessed with a 30-item culturally adapted version of the Domestic and Community Violence Checklist (DCVC, for details see Hermenau et al., 2011; Crombach and Elbert, 2014). It incorporates different experiences of maltreatment from various dimensions (psychological, physical, sexual violence, neglect) ranging from small (e.g., being pinched) to very severe events (e.g., sexual abuse). Again, all items were coded dichotomously and summed up. The sum score represents the number of experiences, ranging from 0 to 30.

#### Current Aggressive Behavior

In order to measure the level of perpetration of violence, questions from the DCVC were also asked from a perpetrator’s perspective (e.g., have you pinched someone) during the period of the last 3 months. Three items were added to assess reactive components of current aggression (e.g., Have you fought back, because you were attacked), whereas one item was removed (having witnessed sexual abuse), as this was not transformable into a perpetrator’s perspective. Items were coded in the same manner and summed up as above, reaching a sum score from 0 to 32.

#### Appetitive Aggression

To assess the extent of propensity toward appetitive aggression the Appetitive Aggression Scale (AAS) was used (Weierstall and Elbert, 2011). It contains 15 items about the positive and exhilarating perception of violence related to a combatant setting (e.g., “Is it exciting for you if you make an opponent really suffer?”) and were rated on a five-point Likert scale ranging from 0 (I totally disagree) to 4 (I totally agree). Items were summed up to create a sum score between 0 and 60. Internal consistency was very high in the current study (Cronbach’s α = 0.95)

#### PTSD Symptom Severity

The PTSD Symptom Scale-Interview (PSS-I, Foa et al., 1993; Foa and Tolin, 2000) was used to assess the frequency of PTSD symptoms. It is comprised of 17 items, each referring to one of the symptoms for PTSD according to DSM-IV (American Psychiatric Association [APA], 2000). Answers are scored on a four-point Likert scale ranging from 0 (not at all) to 3 (five or more times per week/almost always). Items are summed up to a sum score ranging between 0 and 51. The PSS-I has proven validity in comparable samples (Ertl et al., 2010) and good psychometric properties (Foa and Tolin, 2000). Internal consistency in the current study was high (Cronbach’s α = 0.94).

### Data Analysis

SPSS 21.0 was used for statistical analysis. A MANOVA, followed by alpha-adjusted univariate *F-*tests, was calculated to assess group differences regarding *childhood violence, traumatic events, perpetrated event types, PTSD symptom severity*, and *appetitive aggression*. *Post hoc* tests were conducted using Games-Howell. To assess predictors of *current aggressive behavior* hierarchical multiple linear regression analyses were conducted. Group membership was dummy-coded with combatants as reference group.

No univariate or multivariate outliers were found. Skewness and curtosis of variables as well as homogeneity of variances among groups gave no rise for concern. Pillai’s criterion was used in the MANOVA due to its robustness against violations of homogeneity of covariance. The residuals of the regression analysis were normally distributed and independent, assumptions of homoscedasticity and linearity met. Multicollinearity was of no concern. All analyses were two-tailed and based on α = 0.05 level of significance.

## Results

### Group Differences in Outcomes Related to Trauma and Aggression

**FIGURE 1:**
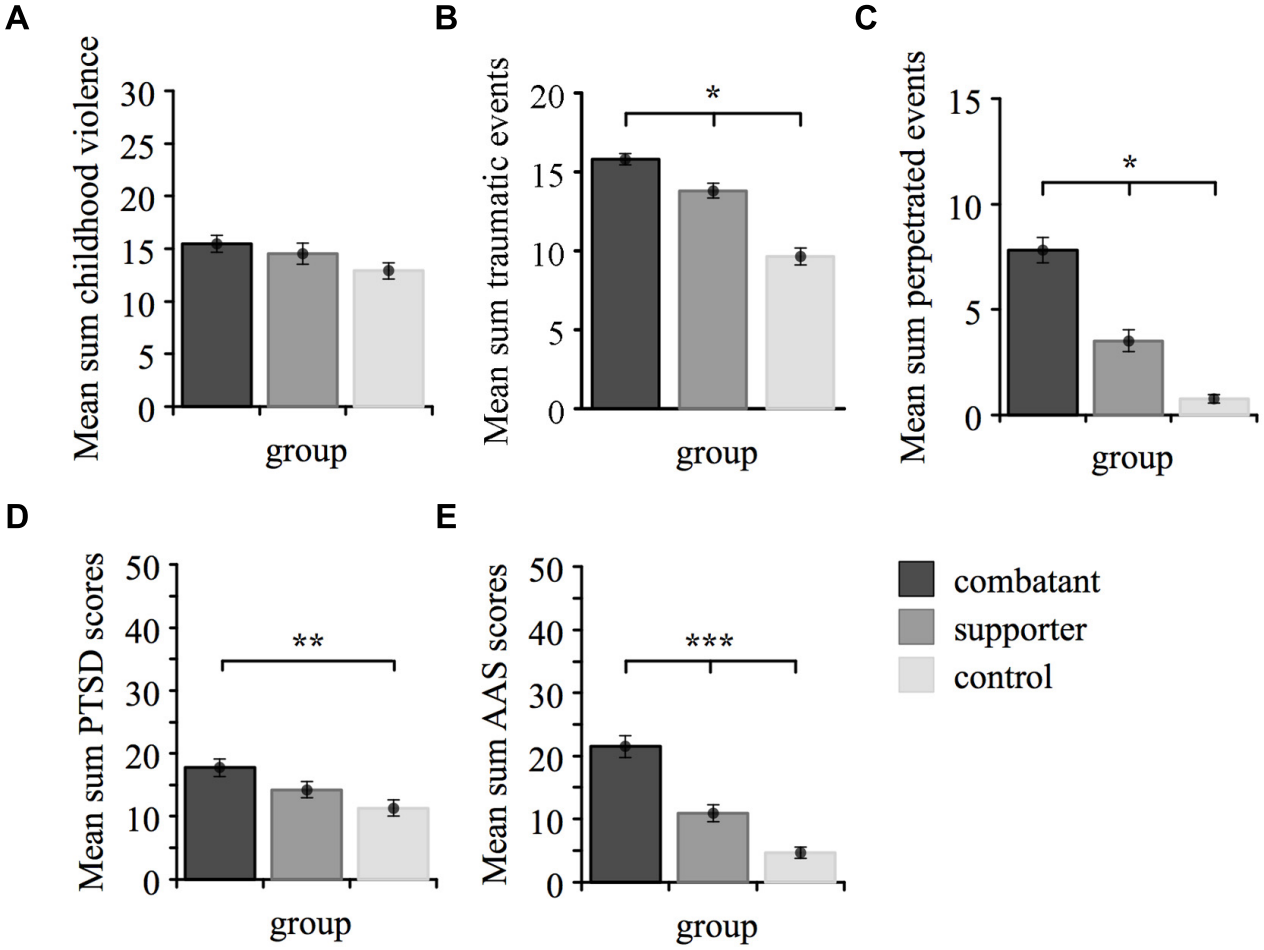
**Differences between the three groups regarding **(A)** childhood violence, **(B)** traumatic event types, **(C)** perpetrated event types, **(D)** posttraumatic stress disorder (PTSD) symptom severity and **(E)** appetitive aggression.** Mean sum scores are plotted on the ordinate, whereas each bar refers to a group. ^∗^*p* < 0.05, ^∗∗^*p* < 0.01, ^∗∗∗^*p* < 0.001.

Using Pillai’s trace, MANOVA showed a significant multivariate effect of group membership, *V* = 0.54, *F*(8,298) = 13.86, *p* < 0.001. Univariate *F*-tests revealed significant group differences with large effect sizes in *traumatic event types, F*(2,152) = 46.92, *p* < 0.001, $\eta_{p}^{2}$ = 0.38, *perpetrated event types, F*(2,152) = 59.01, *p* < 0.001, $\eta_{p}^{2}$ = 0.44 and *appetitive aggression, F*(2,152) = 41.17, *p* < 0.001, $\eta_{p}^{2}$ = 0.35. Former combatants had significantly higher rates compared to both former supporters and controls. A group difference with moderate effect size was found for *PTSD symptom severity, F*(2,152) = 5.96, *p* < 0.01, $\eta_{p}^{2}$ = 0.07. Combatants suffered more severely from PTSD symptoms than controls, but did not differ from supporters. Groups did not differ regarding *childhood violence, F*(2,152) = 2.38, *p* = 0.1. The results of *post hoc* comparisons are illustrated in **Figure 1**.

### Prediction of Current Violent Behavior

As illustrated in **Table 2**, trauma-related variables (traumatic event types, childhood violence, PTSD symptom severity) were moderately associated. Appetitive aggression and perpetrated event types showed a strong association. Overall, trauma- and aggression-related variables correlated with moderate to high size.

**Table 2:** Correlations between outcome variables.

Variable	Childhood violence	Traumatic event types	Perpetrated event types	Posttraumatic stress disorder (PTSD) symptom severity	Appetitive aggression	Current aggressive behavior
Childhood violence	1					
Traumatic event types	0.48^∗∗∗^	1				
Perpetrated event types	0.36^∗∗∗^	0.64^∗∗∗^	1			
PTSD symptom severity	0.47^∗∗∗^	0.54^∗∗∗^	0.45^∗∗∗^	1		
Appetitive aggression	0.19^∗^	0.53^∗∗∗^	0.81^∗∗∗^	0.4^∗∗∗^	1	
Current aggressive behavior	0.51^∗∗∗^	0.41^∗∗∗^	0.49^∗∗∗^	0.34^∗∗∗^	0.44^∗∗∗^	1

*^∗^p < 0.05, ^∗∗∗^p < 0.001.*

To assess predictors for current aggressive behavior, in a first step the two dummy-coded variables of *group membership* variables were entered [*F*(2,152) = 3.77, *p* = 0.025, *R*^2^_adjusted_ = 0.04]. Belonging to the group of controls (*p* < 0.01) or supporters (*p* = 0.09) was associated with lower levels of current aggressive behavior compared to combatants. After adding *appetitive aggression* and *PTSD symptom severity* as predictors in the second step, there was a significant model improvement [*F*(2,150) = 18.01, *p* < 0.001, *R*^2^_adjusted_ = 0.21]. As illustrated in **Table 3**, both, *PTSD symptom severity* and *appetitive aggression* were positively related to current aggressive behavior. Group membership proved insignificant After including *childhood violence*, the model substantially improved in terms of variance explained [*F*(1,149) = 40.03, *p* < 0.001, *R*^2^_adjusted_ = 0.37]. Higher levels of both *appetitive aggression* and *childhood violence* were significantly related to current aggressive behavior. However, *PTSD symptom severity* now failed to reach significance. Adding interaction terms did not improve the model in terms of higher variance explained, nor did they reach significance.

**Table 3:** Multiple regression analysis for the prediction of current aggressive behavior.

	*b*	SE B	β	*t*
**Step 2**				
Dummy control	0.94	1.03	0.09	0.91
Dummy supporter	0.52	0.94	0.05	0.55
Appetitive aggression	0.17	0.04	0.41	4.43^∗∗∗^
PTSD symptom severity	0.10	0.04	0.20	2.51^∗^
**Step 3**				
Dummy control	1.29	0.92	0.13	-1.4
Dummy supporter	0.5	0.84	0.05	0.6
Appetitive aggression	0.17	0.03	0.43	5.16^∗∗∗^
PTSD symptom severity	-0.01	0.04	-0.02	-0.25
Childhood violence	0.36	0.06	0.46	6.33^∗∗∗^

*N = 155; SE, standard error; combatants were used as reference group. The constant is not shown due to better readability; ^∗^*p* < 0.05, ^∗∗∗^*p* < 0.001.*

## Discussion

In the present study former combatants reported higher exposure to traumatic events and greater involvement in lifetime perpetration of aggressive acts compared to both former supporters and civilians. In accordance with the *building block effect*, symptoms of PTSD were also highest among former combatants. Being an active agent of warfare resulted in greater damage of trauma-related mental health in comparison to those who had witnessed war-related actions or were victims of the civil war. These results coincide with previously published studies on former male combatants presenting high impairment due to PTSD (e.g., Weierstall et al., 2013a; Nandi et al., 2015) and on psychological problems in former girl soldiers (Annan et al., 2011).

Severe physical punishment as well as high rates of sexual abuse are typical experiences for girls when growing up in armed groups (McKay and Mazurana, 2004). As more than 50% joined the armed groups when they were younger than 18, they were at particular risk for exposure to high levels of violence during childhood. However, the individuals in the three groups did not differ regarding their exposure to adverse experiences during childhood. A possible explanation could be that domestic violence and corporal punishment are a widespread phenomenon within the Burundian society. Thus, all females are likely to have experienced severe forms of childhood maltreatment. Moreover, due to the civil war and irrespectively of their own involvement many girls grew up in disrupted families and adverse conditions.

Regarding appetitive aggression, the highest levels were present among former female combatants even several years after the end of the civil war. Compared to civilians, elevated levels of appetitive aggression were also found in supporters. A recent study on Burundian former male combatants and active soldiers identified both exposure to traumatic events and lifetime involvement in violent offenses as principal risk factors for appetitive aggression (Nandi et al., 2015). These factors might also account for differences in appetitive aggression between former female supporters and civilians, as the former reported greater exposure to traumatic events and perpetration of violent acts. Moreover, as self-committed violence is known to be strongly correlated with appetitive aggression (e.g., Hecker et al., 2012; Weierstall et al., 2013b) previously found differences between males and females (Weierstall et al., 2011) are likely to originate from a substantial gender difference in exposure to warfare. In all war scenarios, male combatants are exposed to combat more frequently and for a longer period of time, resulting in a higher probability to be shaped by these environments. In favor of this interpretation are the results of Meyer-Parlapanis et al. (submitted) with matched samples and thus comparable levels of both exposure to traumatic events and perpetration of violence. However, it may well be possible that men are more easily attracted to participate in armed conflicts whereas this may be true only for a selection of women. Our results demonstrate, that those women who are exposed to fighting and combat, develop levels of appetitive aggression similar to their male counterparts (Weierstall et al., 2013a; Meyer-Parlapanis et al., submitted) which can be considered as stable and long-term adaptation to adverse and insecure environments (Crombach and Elbert, 2014).

Furthermore, evidence was provided that appetitive aggression, PTSD symptoms and violence experienced during childhood are important factors explaining the perpetration of everyday violence. Consistent to previous research, appetitive aggression appears to be crucial in thriving, ongoing violent behavior in post-conflict regions (Crombach and Elbert, 2014), highlighting the importance to focus on in demobilization processes of former combatants. In contrast to appetitive aggression, PTSD and violence experienced during childhood seem to influence aggressive behavior via the same mechanism, indicated by the fact that the former lost its predictive value as soon as the latter was included in the regression model. Research has found PTSD symptoms of hyperarousal to be associated with deficits in self-regulatory competences (Galovski and Lyons, 2004; Orth and Wieland, 2006; Tull et al., 2007). Also a history of childhood abuse is related to a lack of the development of behavioral control strategies (Brodsky et al., 2001; Roy, 2005). On a neurobiological level, both are linked to altered neurobiological brain circuitries (Elbert et al., 2006; Kolassa and Elbert, 2007; Braquehais et al., 2010), which partially overlap with neurobiological changes found in persons with low behavioral control strategies (Braquehais et al., 2010). These alterations in the neural circuitry due to impeded regulatory competences predispose individuals to react aggressively (Davidson, 2000; DeWall et al., 2007) and are likely to be the underlying mechanism of childhood maltreatment outperforming PTSD in predicting current aggressive behavior. Hereby, primarily anger-driven reactive forms of aggression are affected, whereas appetitive aggression presents a distinct brain pattern (Moran et al., 2014). Thus, appetitive aggression and childhood maltreatment independently contribute to different forms of aggression resulting in an overall enhanced level of aggressive acts further promoting the cycle of violence.

The current study has some limitations. Relying on self-report, memory effects and social desirability might have biased reporting appetitive aggression and aggressive acts, possibly underestimating their impact. Furthermore, the context in which violent acts occurred was not assessed. In a traditional patriarchal society such as Burundi it is likely that the females perpetrated a significant part of violence against their own children, promoting a trans-generational cycle of violence (Roth et al., 2014; Crombach and Bambonyé, 2015). However, females reported both domestic and community violence, hence posing a threat to the overall development of a peaceful society. A more detailed assessment of the context might have yielded information about who is affected most by enhanced female aggressiveness.

The retrospective cohort-design limits interpretations regarding causality and direction of the reported effects. Only the chronological restrictions of the variables within the aggression model render the determined relationships between cause-and-effect plausible. Furthermore, selective dropout might have influenced the results. Severely affected women with high rates of PTSD might not have been able to participate in the interviews. Lastly, buffering effects of the community were not targeted in the current study. It is unclear, if social rejection of former fighters, who might have violated traditional gender roles, also occurred in the Burundian context. Moreover, the impact of social support, which can serve as a protective factor especially among females (Chaumba and Bride, 2010), has not been approached at all.

## Conclusion

The present study demonstrates that in order to disrupt the cycle of violence in post-conflict regions and to help promote and sustain long lasting peace building processes, it is essential to address adverse childhood experiences, appetitive aggression and trauma-related ill-health in females formerly associated with combat. Moreover, neglecting to provide adequate resources to females in demobilization processes (Schauer and Elbert, 2010) leaves them vulnerable in the challenges related to their victimhood. Living in societies in which women are typically charged with the role of homemaking, these untreated female members of armed groups often transition directly into the roles as caregivers for their children. For those suffering from PTSD symptomology and other mental health complications, trading in guns for cooking spoons invariably places their children at risk for being the next victims in the cycle of violence. Hence prospective studies should be aimed at implementing therapeutic interventions in this particular population, comprising perspectives of both perpetrator and victim. Moreover, the evaluation of their effectiveness in terms of decreasing future aggressive acts and improving mental health is a meaningful next step. Lastly, in order to further disentangle the cycle of violence precedents and risk factors of appetitive aggression should be identified with a focus on potential differences in its development between males and females.

## Conflict of Interest Statement

The authors declare that the research was conducted in the absence of any commercial or financial relationships that could be construed as a potential conflict of interest.
